# Supplementary material for: Energy-efficient extraction of linear alkanes from various isomers using structured metal-organic framework membrane
Source: Nat Commun. 2023 Oct 19;14:6617. doi: 10.1038/s41467-023-42397-x (PMC10587105; doi:10.1038/s41467-023-42397-x)
Supplement: Supplementary file 1 — Supplementary Information [file 41467_2023_42397_MOESM1_ESM.pdf]

Supplementary Information

Energy-efficient extraction of linear alkanes from various isomers using structured metal-organic framework membrane

Yuecheng Wang et al.

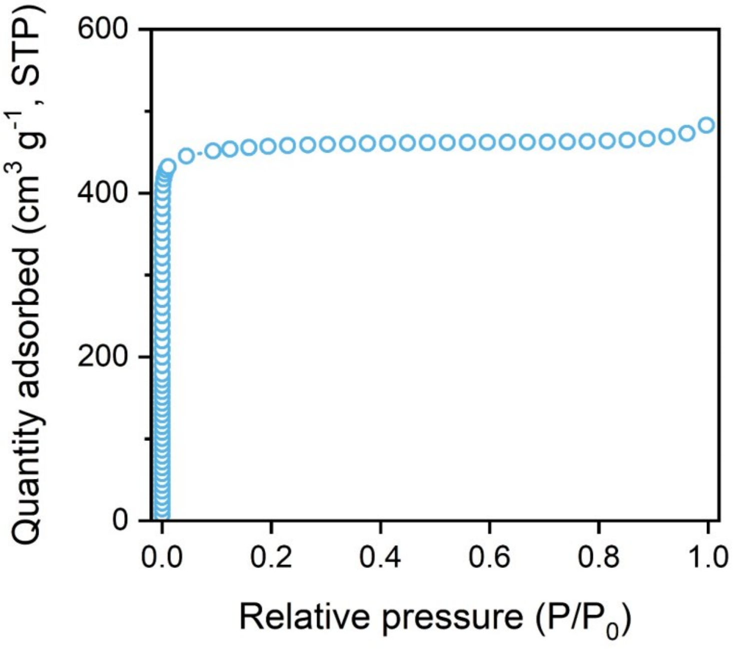

Supplementary Fig. 1 Adsorption isotherm of N_2 on the HKUST-1 powder at 77 K. Source data are provided as a Source Data file.

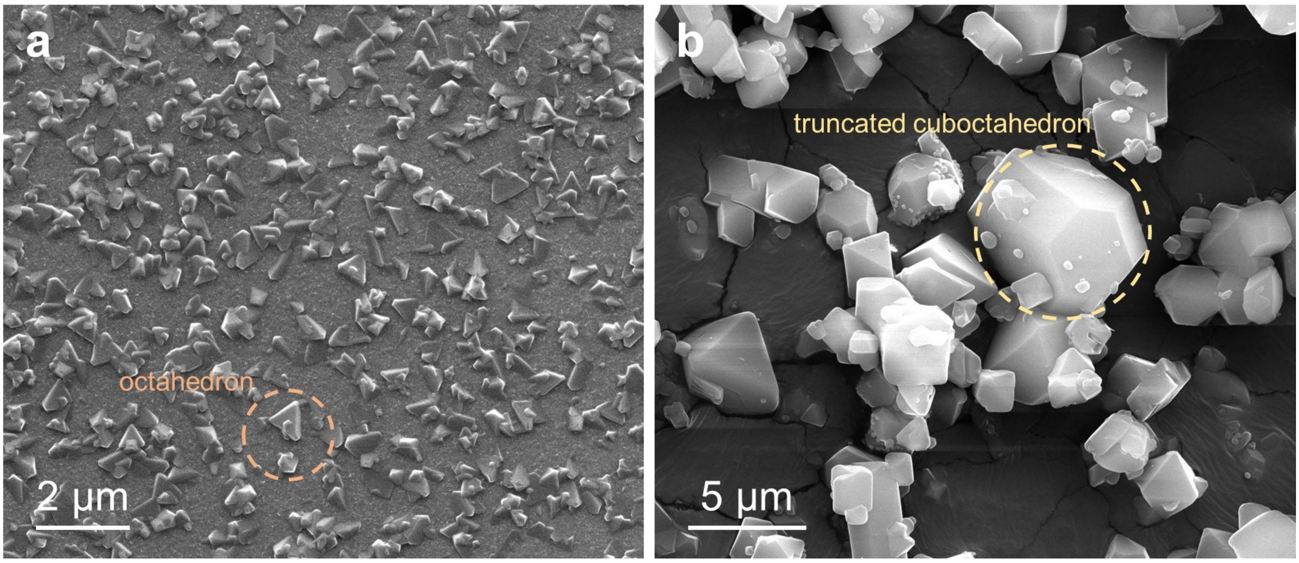

Supplementary Fig. 2 Top-view SEM images of HKUST-1 crystals generated at the surface of the support (a) and collected from the bulk solution (b).

The alumina support was kept in the bulk solution after static seeding for 60 min, sealed in a stainless-steel autoclave and transferred into an oven (150 ^{o}C) for 120 min. Then the support was removed from the solution, washed repeatedly and subjected to the SEM characterization (Supplementary Fig. 2a). HKUST-1 crystals generated at the surface of the support show a sharp octahedral morphology. At the same time, HKUST-1 powder was collected from the same bulk solution, washed thoroughly and subjected to SEM characterization. As shown in Supplementary Fig. 2b, the HKUST-1 powder shows multiple morphologies, typified by a truncated cuboctahedron morphology.

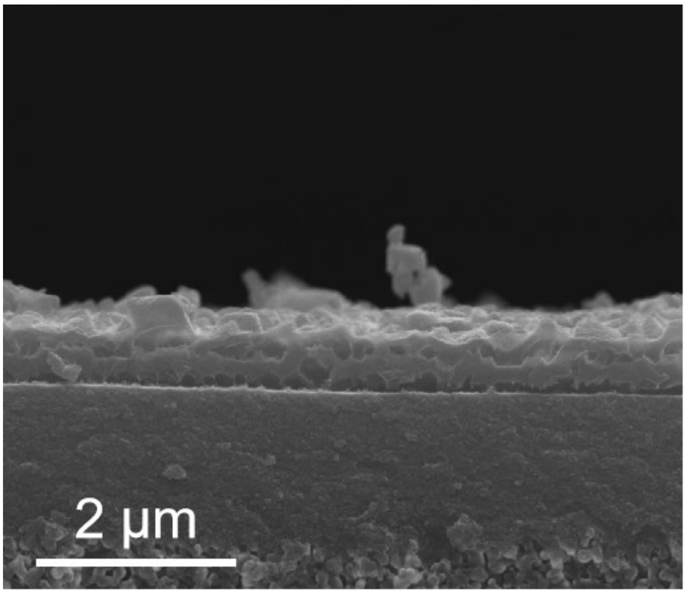

Supplementary Fig. 3 Top-view SEM image of the HKUST-1 membrane without ultrasonication before static seeding.

This is positive evidence to indicate that ultrasonication can help overcome the limitation of the triple phase boundary involving alumina, air (in the pores of alumina) and liquid, expel air in the pores of the alumina support and make nutrients completely wet the support. Without ultrasonication, there would be a lot of hole defects in the membrane layer and between the membrane layer and the support because of the air escape from the pores of the support during membrane preparation.

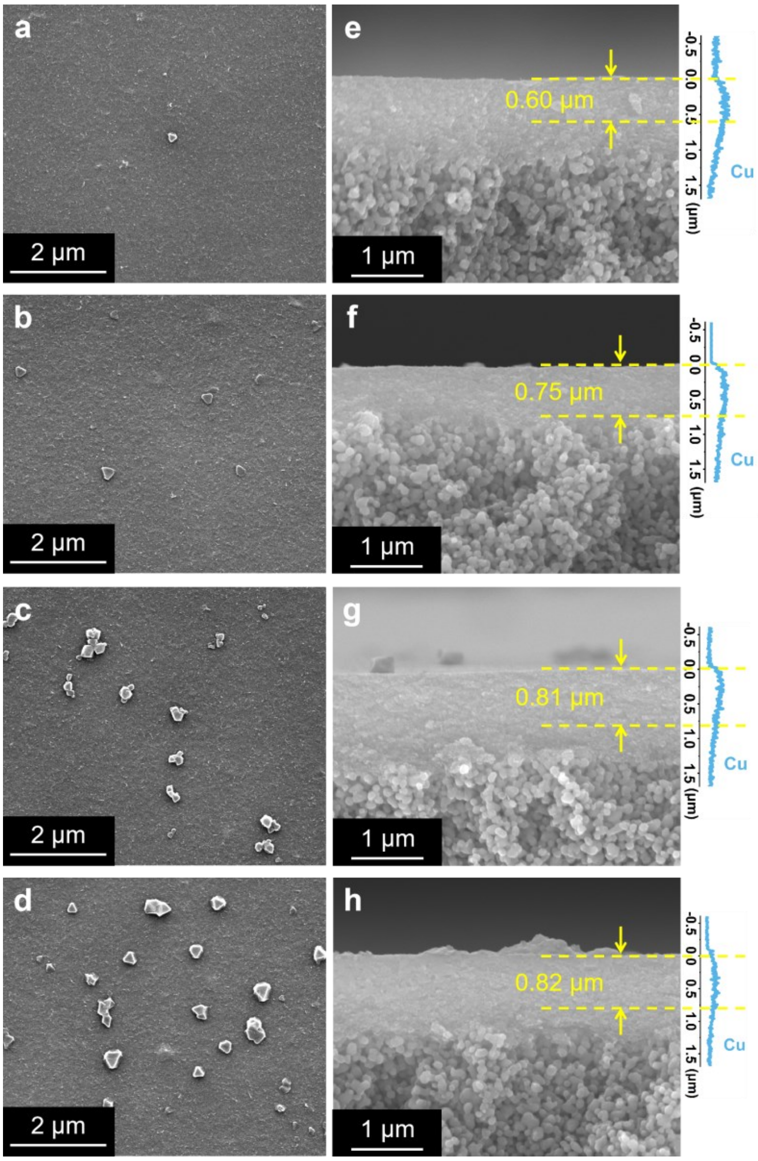

Supplementary Fig. 4 Top-view (a-d) and cross-sectional SEM images coupled with the copper distribution along the depth (e-h) of the Al_2O_3 support after static seeding for 30-120 min. a and e, 30 min. **b and f**, 60 min. **c and g**, 90 min. **d and h**, 120 min.

The alumina disk after static seeding was washed in DMF under stirring for 12 h to remove Cu^{2+} adsorbed in the support. DMF was replaced every 4 h.

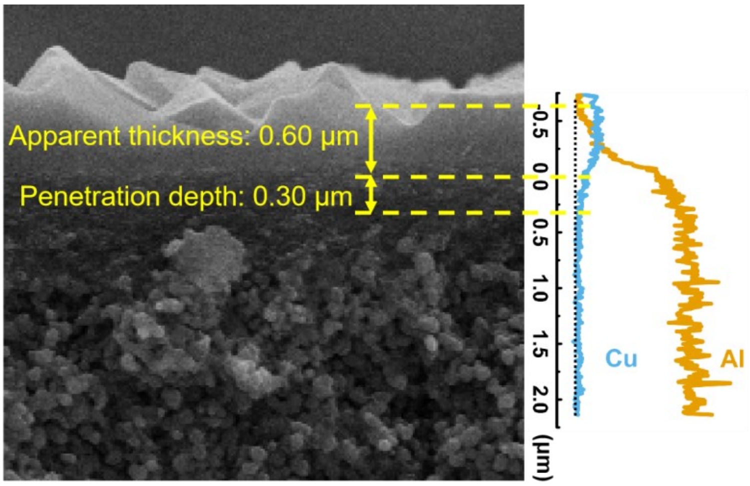

Supplementary Fig. 5 Cross-sectional SEM image of the HKUST-1 membrane (1 SS-TC cycle) and the corresponding distribution of alumina and copper along the depth.

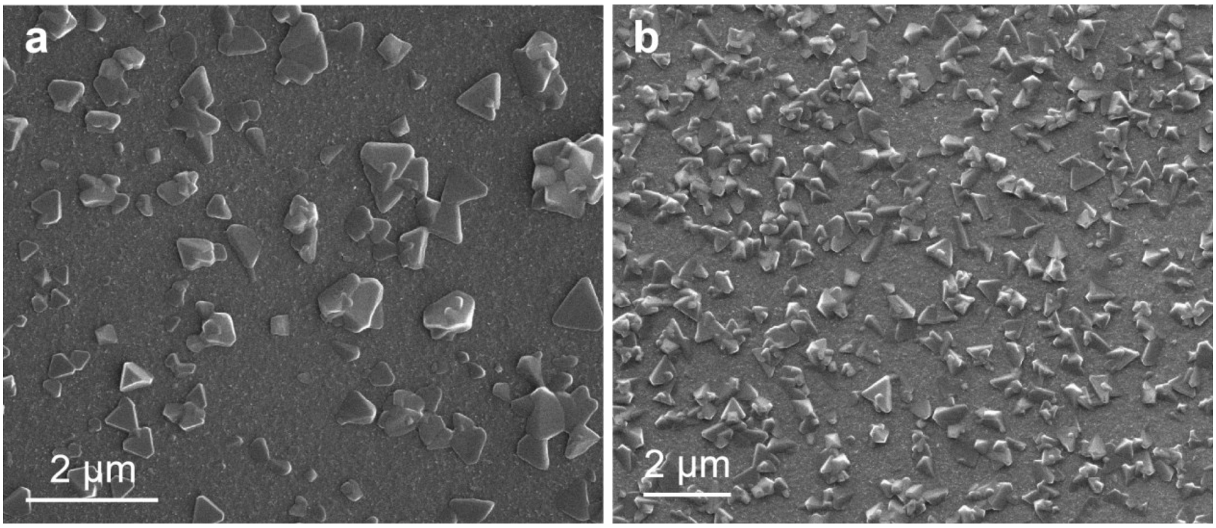

Supplementary Fig. 6 Top-view SEM images of the HKUST-1 membrane in two groups of comparative studies. a, The alumina support that was immersed into a fresh precursor solution in a stainless-steel autoclave and immediately transferred into an oven (150 ^{o}C) to implement a solvothermal reaction for 120 min. **b,** The alumina support that was kept in the bulk solution after static seeding for 60 min, sealed in a stainless-steel autoclave and transferred into an oven (150 ^{o}C) for 120 min.

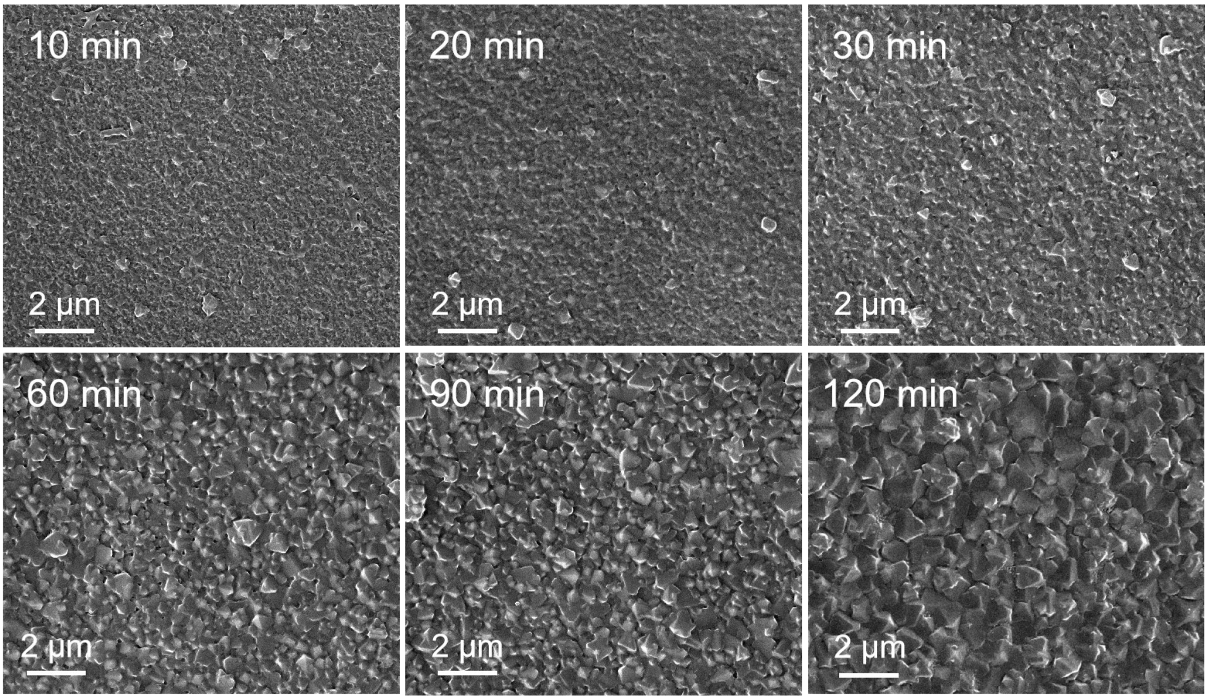

Supplementary Fig. 7 Top-view SEM images of the HKUST-1 membrane with different thermal conversion times.

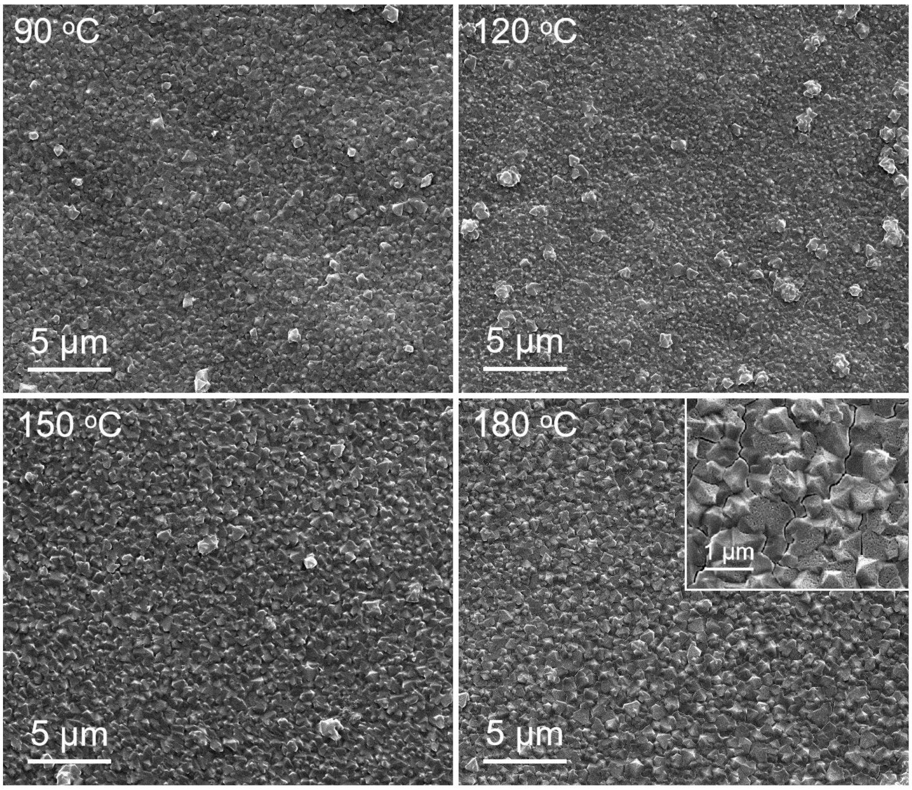

Supplementary Fig. 8 Top-view SEM images of the HKUST-1 membrane with different thermal conversion temperatures.

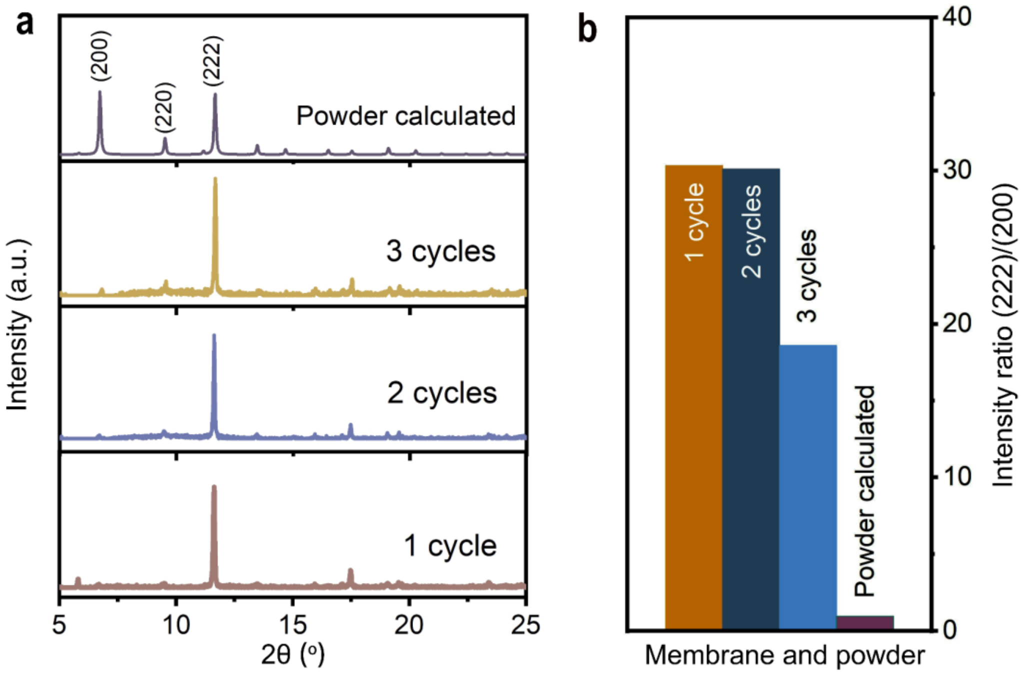

Supplementary Fig. 9 Orientation of HKUST-1 membranes after different SS-TC cycles. a, XRD patterns of membranes after different SS-TC cycles. **b**, The intensity ratio (222)/(200) of the membranes determined by experimental patterns and the standard powder calculated based on the structure CCDC 755080. Source data are provided as a Source Data file.

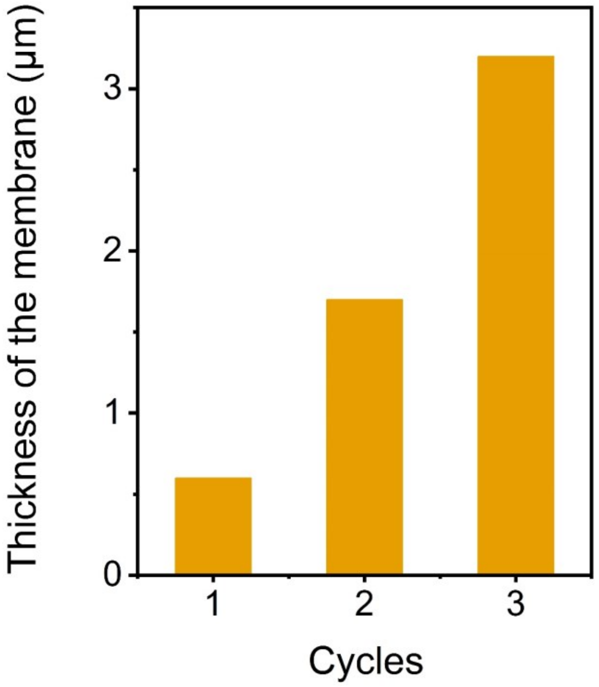

Supplementary Fig. 10 Variation in the thickness of HKUST-1 membranes after different SS-TC cycles. Source data are provided as a Source Data file.

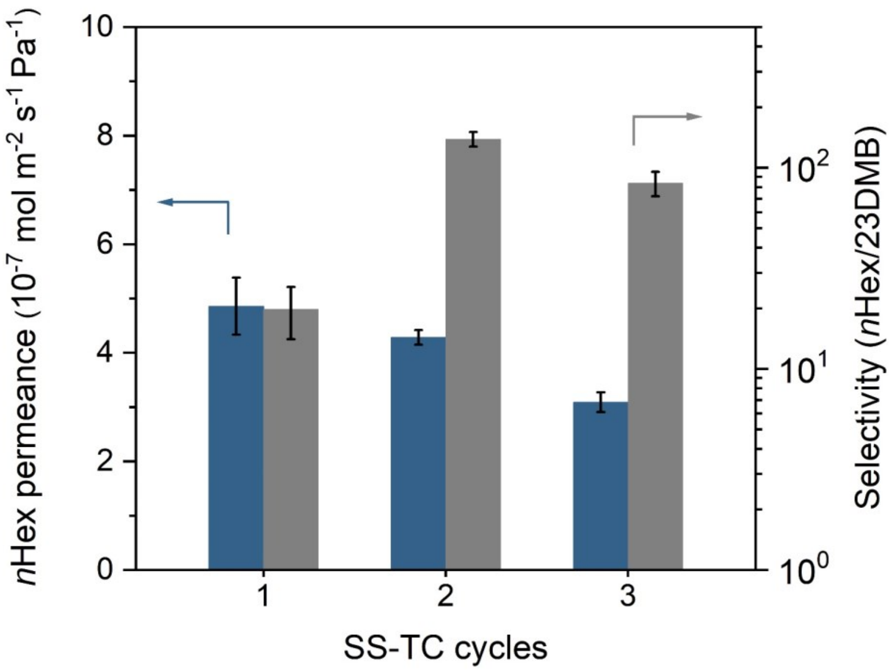

Supplementary Fig. 11 Separation performances of HKUST-1 membranes after different SS-TC cycles. The feed solution is 10/90 *n*Hex/23DMB at 30 °C. Error bars represent the standard deviation of the results from three membranes. Source data are provided as a Source Data file.

The orientation and thickness of the membranes are shown in Supplementary Fig. 9 and Fig. 10. After 2 cycles of SS-TC, the thickness of the membrane was increased to 1.7 μm whereas the intensity ratio (222)/(200) was barely changed, concurrent with a significant improvement in the separation selectivity for *n*Hex/23DMB. We speculate that 2 cycles of SS-TC can eliminate minor local defects in the membranes completely, thus leading to a significant improvement in the isomer separation accuracy. After 3 cycles of SS-TC, the intensity ratio (222)/(200) decreased to 18.6, suggesting that the proportion of {100} crystal facets aligned out-of-plane was not negligible. The molecular sieving property was thus softened, corresponding to a reduction in the selectivity for *n*Hex/23DMB.

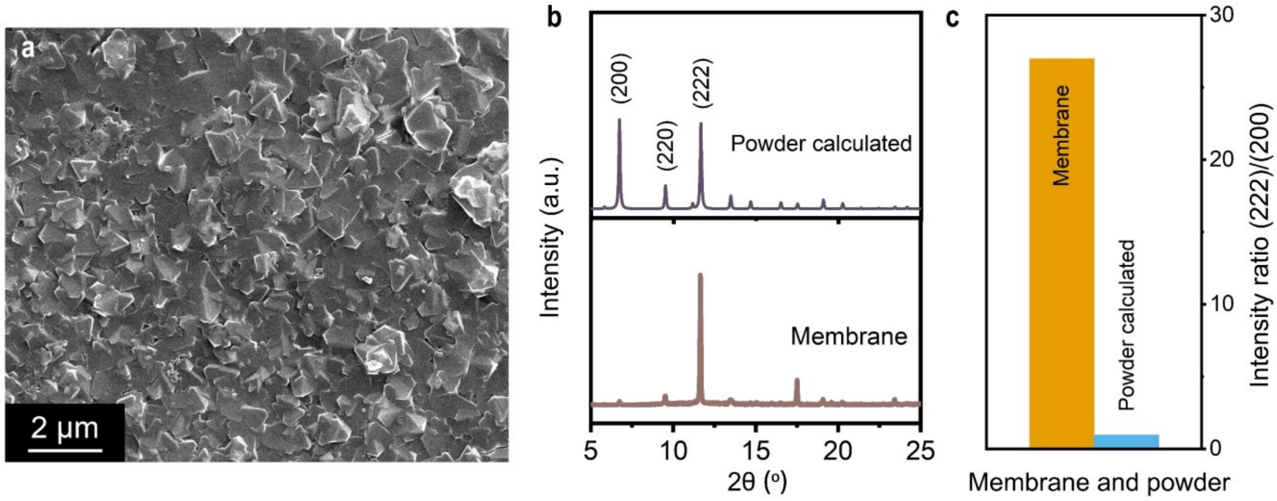

Supplementary Fig. 12 The HKUST-1 membrane prepared by the SS-TC process, in which the thermal conversion process was sealed in a closed system. a, Top-view SEM images of the membrane. **b**, XRD patterns of the membrane. **c**, The intensity ratio (222)/(200) of the membranes determined by experimental patterns and the standard powder calculated based on the structure CCDC 755080. Source data are provided as a Source Data file.

The selectivity of the HKUST-1 membrane for the 10/90 *n*Hex/23DMB feed solution is 122, which is comparable to the membrane prepared in an open vessel.

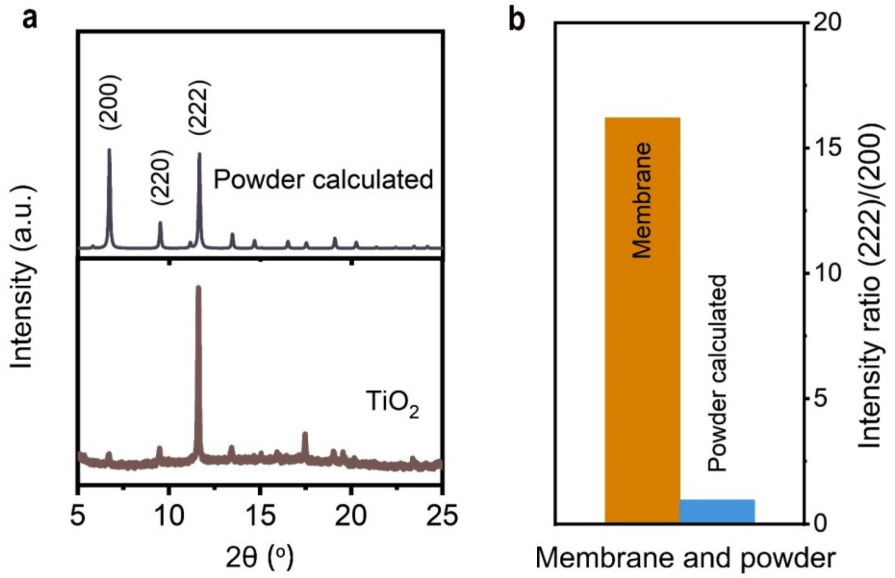

Supplementary Fig. 13 The HKUST-1 membrane prepared on the TiO_2 (5 nm pores) support after 2 SS-TC cycles. **a**, XRD patterns of the HKUST-1 membranes. **b**, The intensity ratio (222)/(200) of the membranes determined by experimental patterns and the standard powder calculated based on the structure CCDC 755080. Source data are provided as a Source Data file.

The HKUST-1 membrane prepared on the TiO_2 support shows 4.10×10^{-7} mol m^{-2} s^{-1} Pa^{-1} for the permeance of *n*Hex and 52.6 for the selectivity of *n*Hex/23DMB.

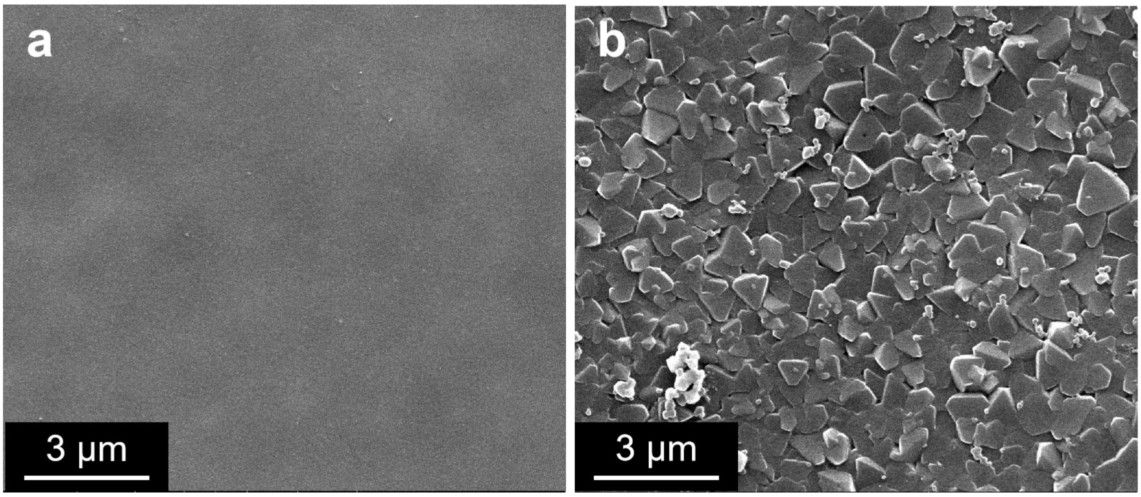

Supplementary Fig. 14 Top-view SEM images of the bare TiO_2 (a), and HKUST-1 membrane prepared on the TiO_2 support after 2 SS-TC cycles (b).

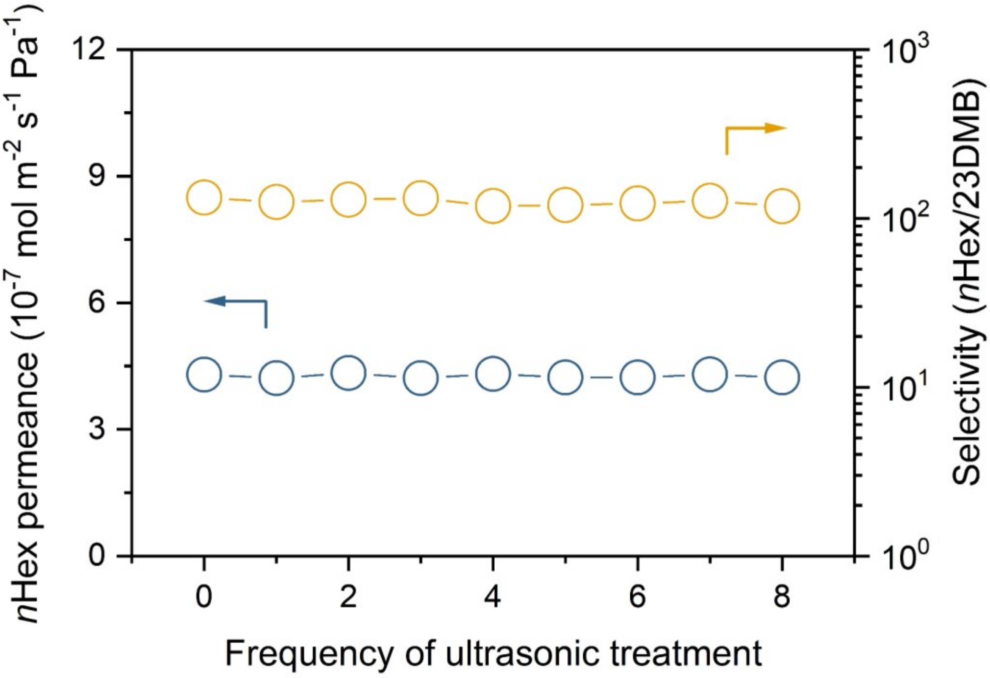

Supplementary Fig. 15 Separation properties toward the 10/90 *n*Hex/23DMB feed solution with the frequency of the ultrasonic treatment. Source data are provided as a Source Data file.

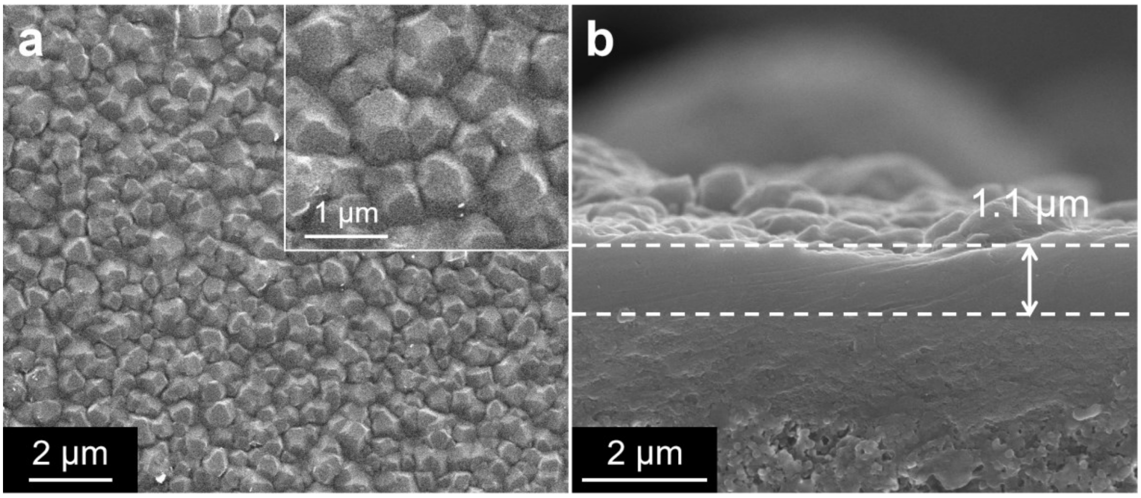

Supplementary Fig. 16 Top-view and cross-sectional SEM images of the near-random-oriented HKUST-1 membrane.

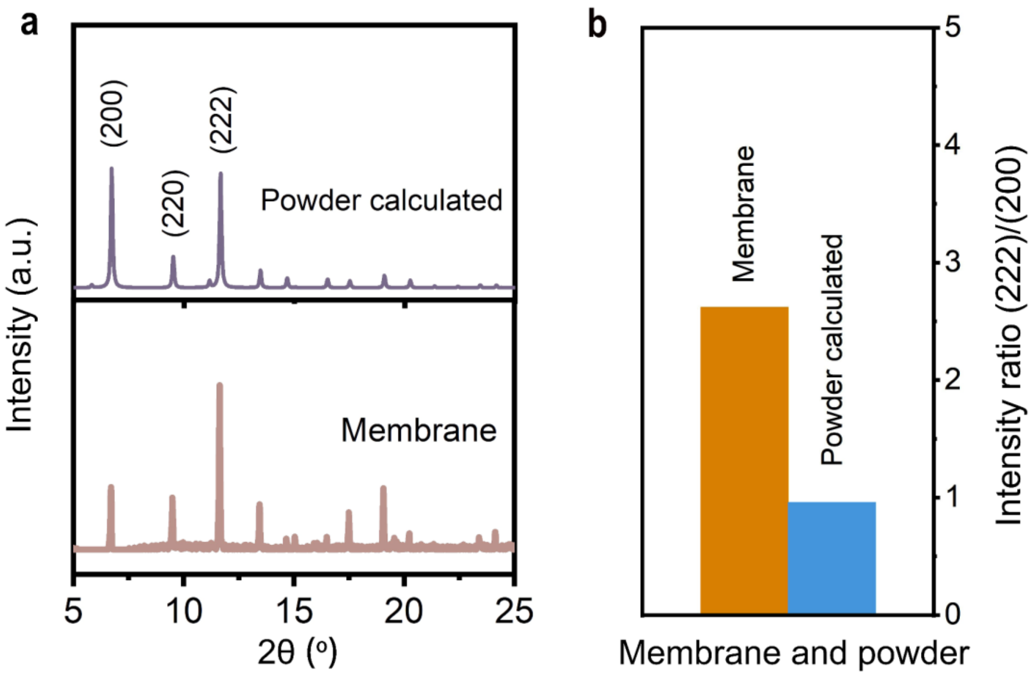

Supplementary Fig. 17 The near-random-oriented HKUST-1 membrane proved by XRD. a, XRD patterns of the membrane. **b,** The intensity ratio (222)/(200) of the membranes determined by experimental patterns and the standard powder calculated based on the structure CCDC 755080. Source data are provided as a Source Data file.

a

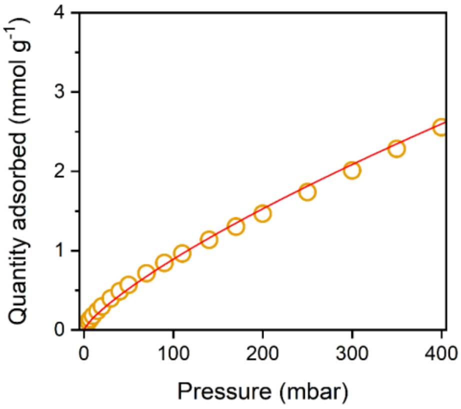

Model	Langmuire- Freundlich
Equation	y=(a*b*x^(1-c))/(1+b*x^(1-c))
a	75.44875 ± 61.48144
b	3.19675E-4 ± 2.24374E-4
c	0.21304 ± 0.02586
Adj. R-Square	0.99906

b

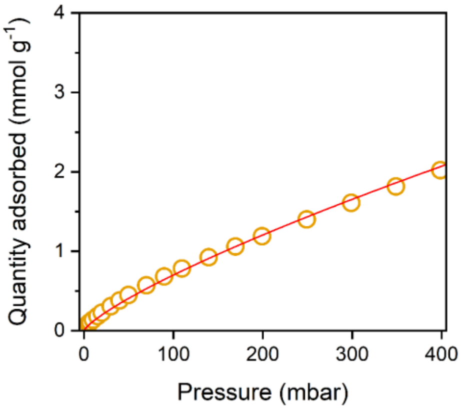

Model	Langmuire- Freundlich
Equation	y=(a*b*x^(1-c))/(1+b*x^(1-c))
a	425.92872 ± 1751.65202
b	4.46102E-5 ± 1.79887E-4
c	0.21617 ± 0.01799
Adj. R-Square	0.99956

Supplementary Fig. 18 Adsorption isotherms of ethane on the HKUST-1 powder. a, 30 °C**. b,** 40 °C**.** Source data are provided as a Source Data file.

a

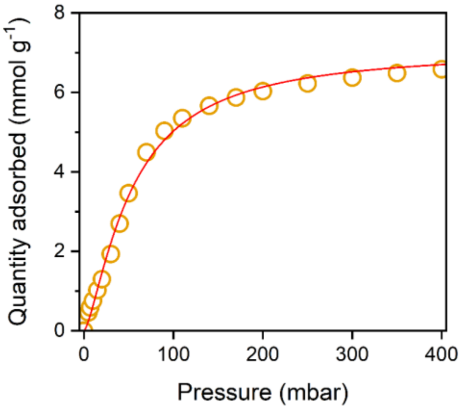

Model	Langmuire- Freundlich
Equation	y=(a*b*x^(1-c))/(1+b*x^(1-c))
a	7.09659 ± 0.05564
b	0.00389 ± 7.1602E-4
c	-0.39753 ± 0.04844
Adj. R-Square	0.99644

b

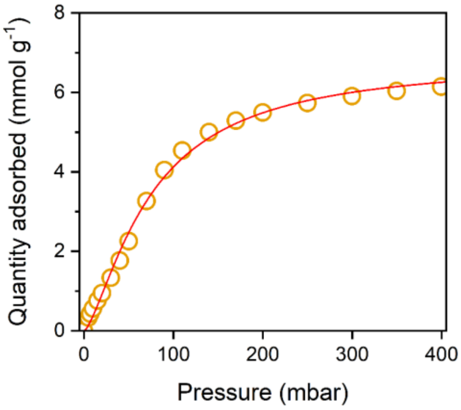

Model	Langmuire- Freundlich
Equation	y=(a*b*x^(1-c))/(1+b*x^(1-c))
a	6.81859 ± 0.05896
b	0.00213 ± 4.16096E-4
c	-0.42792 ± 0.04799
Adj. R-Square	0.99708

Supplementary Fig. 19 Adsorption isotherms of propane on the HKUST-1 powder. a, 30 °C. **b,** 40 °C. Source data are provided as a Source Data file.

a

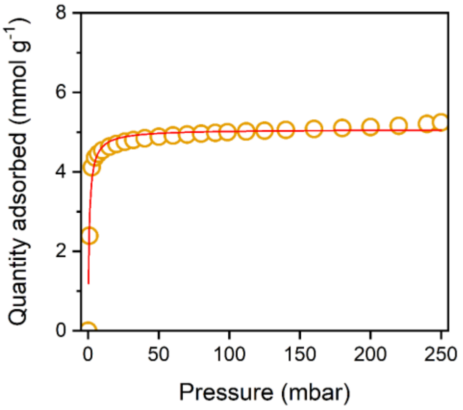

Model	Langmuire- Freundlich
Equation	y=(a*b*x^(1-c))/(1+b*x^(1-c))
a	5.0696 ± 0.04258
b	1.1504 ± 0.09997
c	0.04468 ± 0.08844
Adj. R-Square	0.9491

b

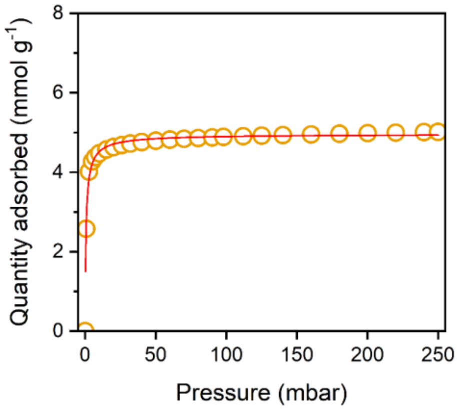

Model	Langmuire- Freundlich
Equation	y=(a*b*x^(1-c))/(1+b*x^(1-c))
a	4.96396 ± 0.02606
b	1.41407 ± 0.06781
c	0.14623 ± 0.0483
Adj. R-Square	0.98013

Supplementary Fig. 20 Adsorption isotherms of *n*Hex on the HKUST-1 powder. a, 30 °C**. b,** 40 °C**.** Source data are provided as a Source Data file.

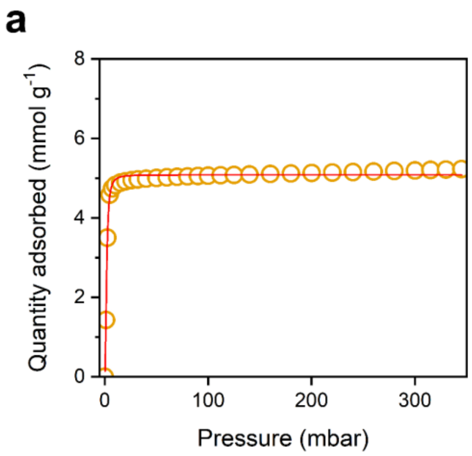

Model	Langmuire- Freundlich
Equation	y=(a*b*x^(1-c))/(1+b*x^(1-c))
a	5.08082 ± 0.01837
b	0.28448 ± 0.02721
c	-1.14872 ± 0.11471
Adj. R-Square	0.984

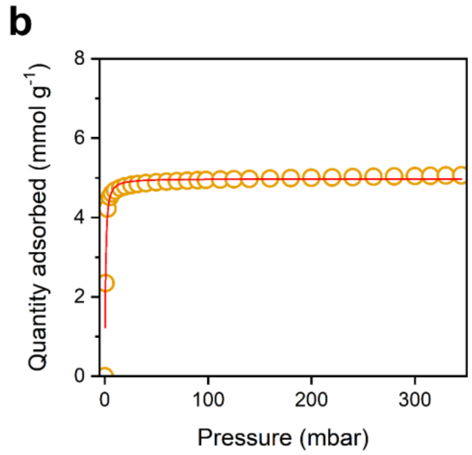

Model	Langmuire- Freundlich
Equation	y=(a*b*x^(1-c))/(1+b*x^(1-c))
a	4.97194 ± 0.01665
b	1.25838 ± 0.06539
c	-0.26683 ± 0.06617
Adj. R-Square	0.97784

Supplementary Fig. 21 Adsorption isotherms of 2MP on the HKUST-1 powder. a, 30 °C. **b,** 40 °C. Source data are provided as a Source Data file.

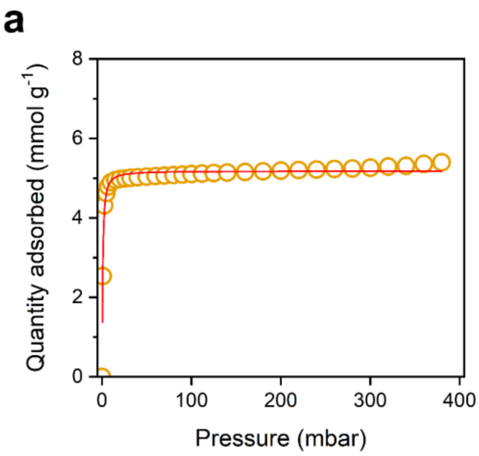

Model	Langmuire- Freundlich
Equation	y=(a*b*x^(1-c))/(1+b*x^(1-c))
a	5.17605 ± 0.02275
b	1.17479 ± 0.08279
c	-0.2259 ± 0.08565
Adj. R-Square	0.95974

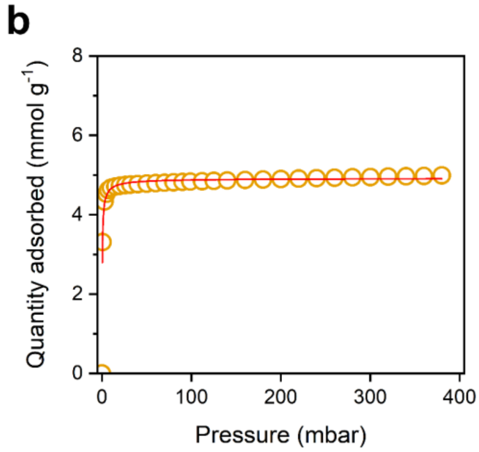

Model	Langmuire- Freundlich
Equation	y=(a*b*x^(1-c))/(1+b*x^(1-c))
a	4.91853 ± 0.01445
b	2.8267 ± 0.11126
c	0.20635 ± 0.04241
Adj. R-Square	0.97507

Supplementary Fig. 22 Adsorption isotherms of 23DMB on the HKUST-1 powder. a, 30 °C. **b,** 40 °C. Source data are provided as a Source Data file.

a

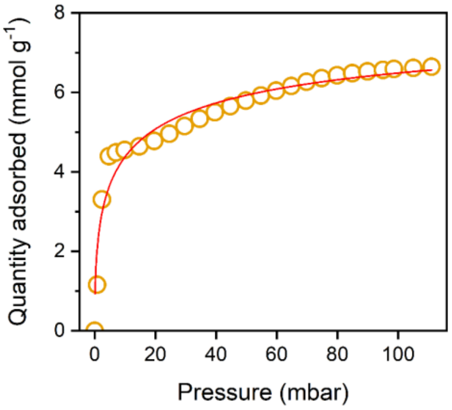

Model	Langmuire- Freundlich
Equation	y=(a*b*x^(1-c))/(1+b*x^(1-c))
a	8.52142 ± 1.23072
b	0.35065 ± 0.05033
c	0.52063 ± 0.10378
Adj. R-Square	0.93432

b

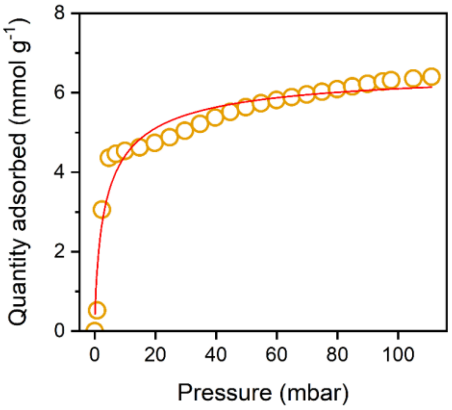

Model	Langmuire- Freundlich
Equation	y=(a*b*x^(1-c))/(1+b*x^(1-c))
a	6.66324 ± 0.40181
b	0.35442 ± 0.06207
c	0.25713 ± 0.13912
Adj. R-Square	0.91449

Supplementary Fig. 23 Adsorption isotherms of *c*Hex on the HKUST-1 powder. a, 30 °C. **b,** 40 °C. Source data are provided as a Source Data file.

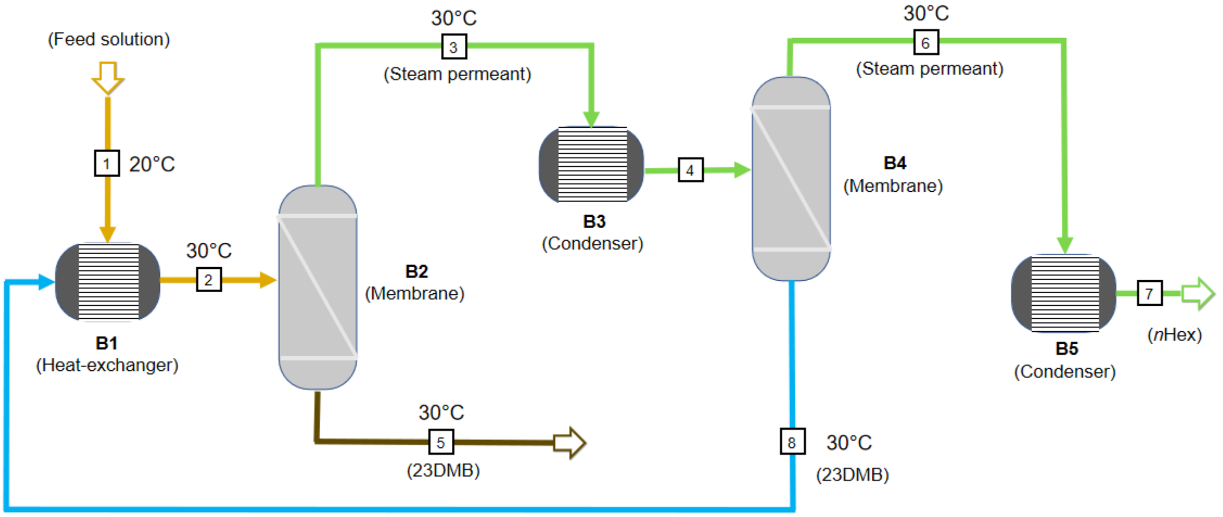

		1	2	3	4	5	6	7	8
Process parameters	Temperature (°C)	20	30	30	30	30	30	-21	30
	Pressure (kPa)	405	200	2	200	405	2	2	200
	Volume flow rate (m^3 h^{-1})	15	15	2	2	13	1	1	1
Energy consumption		Q1		Q2		Q3		Q	
	Energy consumption (GJ per ton of feed solution)	0.0241		0.0927		0.104		0.221	

Supplementary Fig. 24 Simulated industrial roadmap for the separation of 10/90 *n*Hex/23DMB through two-step membrane pervaporation.

Aspen Plus v7.2 software was employed to implement a chemical process simulation for the separation of 10/90 *n*Hex/23DMB with an annual capacity of 10,000 tons. To meet the purity target of 99 wt.% for both *n*Hex and 23DMB, two-step membrane separation operations are needed. Based on the experimental conditions in our lab, the temperature of the raw liquid feed is set at 20 °C. The feed liquid is further heated by B1 to the target temperature (30 °C), and then flows into the membrane module (B2) for pervaporation. The permeate side of the membrane was connected to a vacuum pump, maintaining an absolute pressure of 2 kPa. The permeant vapour is collected in the condenser (B3, equivalent to a cold trap) at a temperature of 30 °C, then flows into the membrane module (B4) for the second pervaporation. The energy consumption of *n*Hex/23DMB separation based on membrane pervaporation is dominated by three factors, namely, the heat load in B1, denoted as $Q1$; the latent heat of *n*Hex vaporization in B2 and B4, denoted as $Q2$; and the cooling load in B3 and B5, denoted as $Q3$. Regardless of electricity in the pervaporation process, the total energy consumption $Q = Q1 + Q2 + Q3$.

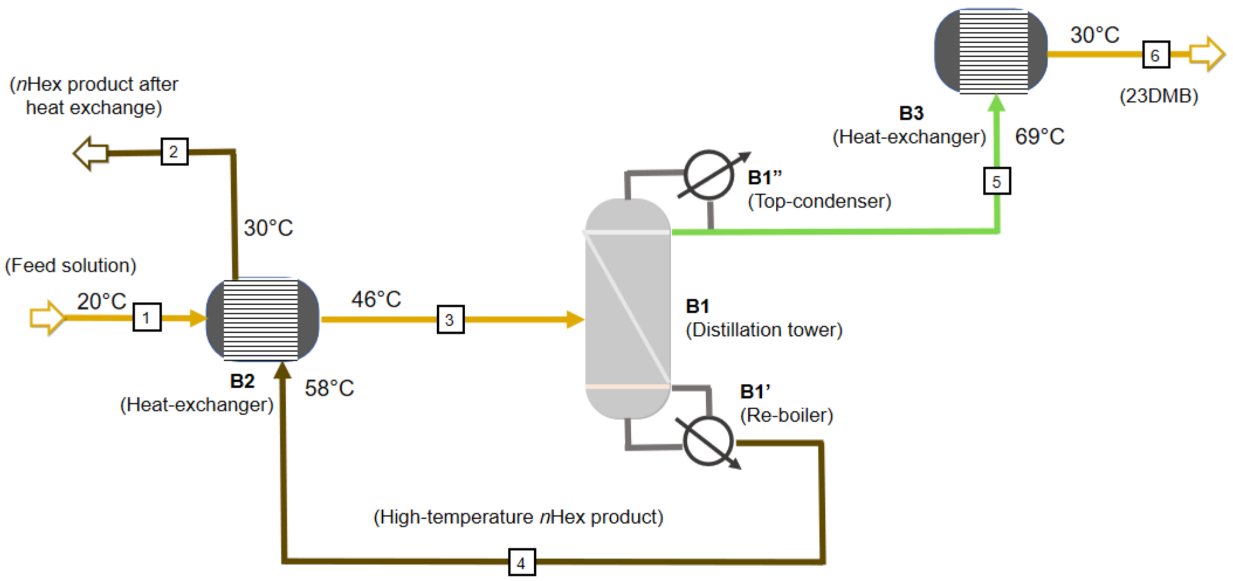

		1	2	3	4	5	6
Process parameters	Temperature (℃)	20	30	46	58	69	30
	Pressure (kPa)	405	101	405	101	101	101
	Volume flow rate (m^3 h^{-1})	15	13	15	13	1	1
Energy consumption		Q1		Q2		Q	
	Energy consumption (GJ per ton of feed solution)	1.17		1.15		2.32	

Supplementary Fig. 25 The simulated industrial roadmap for the separation of 10/90 *n*Hex/23DMB through vacuum distillation.

Aspen Plus v7.2 software was employed to implement a chemical process simulation for the separation of 10/90 *n*Hex/23DMB with an annual capacity of 10,000 tons. The temperature of the raw *n*Hex/23DMB solution was set at 20 °C, and its flow rate was set at 15 m^3 h^{-1}. The preheated raw liquid feed flows into the distillation tower (B1) for vacuum distillation. Heat transfer occurs in heat-exchanger B2 between the raw liquid feed and the *n*Hex product collected at the bottom of the tower. The ultimate temperature of high-purity 23DMB drops to 30 °C. The 23DMB phase is collected at the top of the tower, where the pressure and temperature are -101 kPa and 69 °C, respectively, and *n*Hex is collected at the bottom of the tower at a temperature of 58 °C. The heat load in the bottom re-boiler (B1') is denoted as *Q1*, and the cooling load in the top-condenser (B3'') is denoted as *Q2*. Regardless of the electricity in the vacuum distillation, the total energy consumption *Q* = *Q1*+ *Q2*.

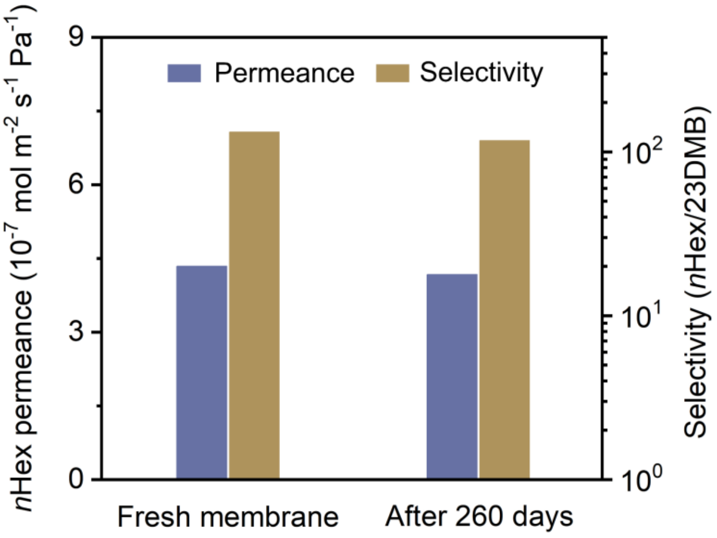

Supplementary Fig. 26 Separation performances of the fresh membrane and the membrane kept in an atmospheric environment for 260 days. The feed solution is 10/90 *n*Hex/23DMB. Source data are provided as a Source Data file.

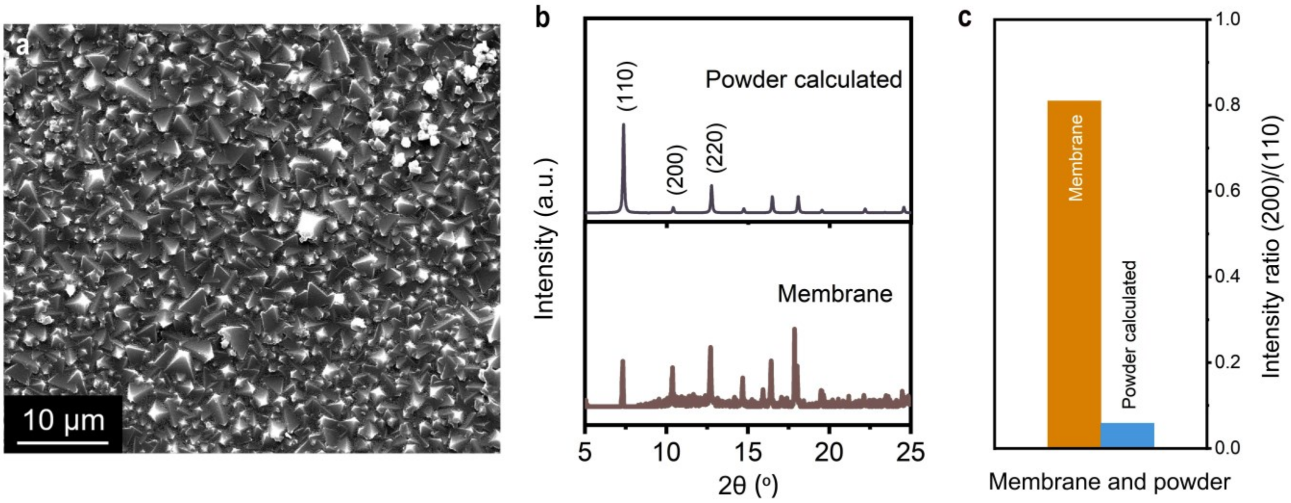

Supplementary Fig. 27 Orientation of the ZIF-8 membrane after 1 SS-TC cycle. a, Top-view SEM images of the oriented ZIF-8 membrane. **b**, XRD patterns of membrane and powder after 1 SS-TC cycle. **c**, The intensity ratio (200)/(110) of the membranes determined by experimental patterns and standard powder calculated based on the structure CCDC 602542. Source data are provided as a Source Data file.

Supplementary Table 1 Molecular kinetic diameters (MKDs), three-dimensional molecular size and molecular geometry factors (MGFs) of different alkanes.

Alkane	MKD (Å)	Three-dimensional molecular size	Length (Å)	Height (Å)	MGF (Length / Height)
Ethane	3.9	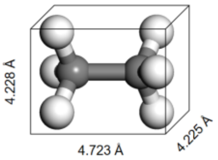	4.723	4.228	1.1
Propane	4.0	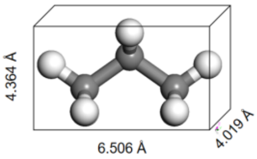	6.506	4.364	1.5
*n*Pen	4.5 [1]	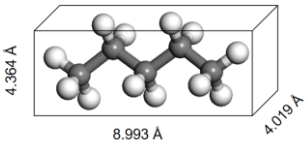	8.993	4.364	2.1
2MB	5.0 [1]	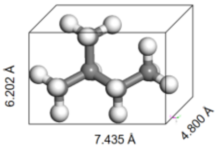	7.435	6.202	1.2
*n*Hex	4.3 [2]	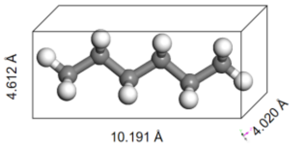	10.191	4.612	2.2
2MP	5.0 [2]	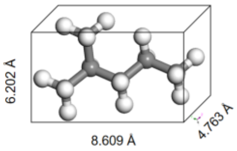	8.609	6.202	1.4
3MP	5.0 [2]	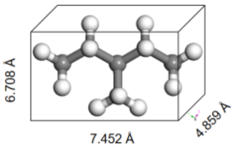	7.452	6.708	1.1
22DMB	6.2 [2]	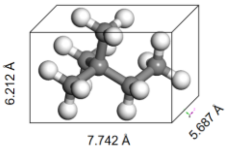	7.742	6.212	1.2
23DMB	5.8 [2]	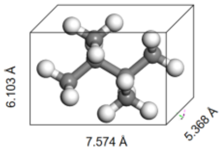	7.574	6.103	1.2
*c*Hex	6.0 [3]	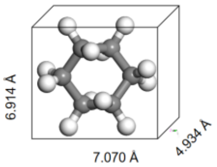	7.070	6.914	1.0

*n*Hep	4.3 [3]	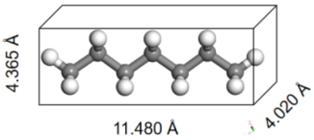	11.480	4.365	2.6
2MH	-	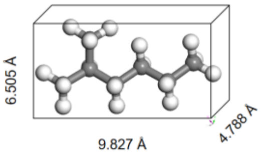	9.827	6.505	1.5
23DMP	-	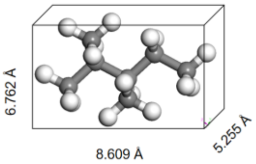	8.609	6.762	1.3

Supplementary Table 2 Single-component permeances of the HKUST-1 membrane.

Single-component permeance (mol m^{-2} s^{-1} Pa^{-1})					
Temperature (K)	C_3H_8	*n*Hex	2MP	23DMB	*c*Hex
303	1.10E-07	1.08E-07	2.38E-08	9.46E-09	9.57E-09
313	1.57E-07	1.64E-07	3.61E-08	1.39E-08	1.40E-08

Supplementary Table 3 Ideal permeation selectivity, adsorption selectivity and diffusion selectivity.

Ideal permeation selectivity					
Temperature (K)	C_3H_8 / *n*Hex	*n*Hex / 2MP	*n*Hex / 23DMB	*n*Hex / *c*Hex	
303	1.02	4.55	11.5	11.3	
313	0.957	4.54	11.8	11.7	
Single-component saturated vapor pressure (mbar)					
Temperature (K)	C_3H_8	*n*Hex	2MP	23DMB	*c*Hex
303	1000	250.0	345.0	379.8	111.0
313	1000	371.8	505.0	551.4	225.5
Adsorption coefficient					
Temperature (K)	C_3H_8	*n*Hex	2MP	23DMB	*c*Hex
303	6.98E-03	2.02E-02	1.47E-02	1.36E-02	5.91E-02
313	6.66E-03	1.97E-02	1.44E-02	1.29E-02	5.53E-02
Adsorption selectivity					
Temperature (K)	C_3H_8 / *n*Hex	*n*Hex / 2MP	*n*Hex / 23DMB	*n*Hex / *c*Hex	
303 K	0.346	1.37	1.48	0.341	
313 K	0.337	1.37	1.53	0.357	
Diffusion selectivity					
Temperature (K)	C_3H_8 / *n*Hex	*n*Hex / 2MP	*n*Hex / 23DMB	*n*Hex / *c*Hex	
303 K	2.94	3.32	7.73	33.2	
313K	2.84	3.31	7.72	32.8	

Supplementary Table 4 Diffusion entropic selectivity and diffusion enthalpic selectivity of *n*Hex over its isomers.

Diffusion entropic selectivity				
Temperature (K)	C_3H_8 / *n*Hex	*n*Hex / 2MP	*n*Hex / 23DMB	*n*Hex / *c*Hex
303	1.02	3.23	7.37	22.7
313	1.02	3.23	7.37	22.7
Diffusion enthalpic selectivity				
Temperature (K)	C_3H_8 / *n*Hex	*n*Hex / 2MP	*n*Hex / 23DMB	*n*Hex / *c*Hex
303	2.87	1.03	1.05	1.46
313	2.77	1.03	1.05	1.45

Supplementary References

1. Wang, H. & Li, J. *Acc. Chem. Res.* **52,** 1968-1978 (2019).
2. Solanki, V. A. & Borah, B. *J. Mater. Chem. A* **123**, 17808-17822 (2019).
3. Zhang, Z., Peh, S. B., Kang, C., Cha, K. & Zhao, D. *Energy Chem* **3**, 100057 (2021).
